# Evaluation of the Effects of Solvents Used in the Fabrication of Microfluidic Devices on Cell Cultures

**DOI:** 10.3390/mi12050550

**Published:** 2021-05-12

**Authors:** Xiaopeng Wen, Seiichiro Takahashi, Kenji Hatakeyama, Ken-ichiro Kamei

**Affiliations:** 1 Institute for Integrated Cell-Material Sciences (WPI-iCeMS), Kyoto University, Yoshida-Ushinomiya-cho, Sakyo-ku, Kyoto 606-8501, Japan; bun.shouhou.4r@kyoto-u.ac.jp; 2 Incubation Center Organs On Chip Project, Ushio INC, 1-6-5 Marunouchi, Chiyoda-ku, Tokyo 100-8150, Japan; s.takahashi@ushio.co.jp (S.T.); k.hatakeyama@ushio.co.jp (K.H.); 3 Wuya College of Innovation, Shenyang Pharmaceutical University, Liaoning 110016, China; 4 Department of Pharmaceutics, Shenyang Pharmaceutical University, Liaoning 110016, China

**Keywords:** microfluidic device, microphysiological systems, organs-on-a-chip, solvent bonding, cytotoxicity, photobonding, vacuum ultraviolet

## Abstract

Microfluidic microphysiological systems (MPSs) or “organs-on-a-chip” are a promising alternative to animal models for drug screening and toxicology tests. However, most microfluidic devices employ polydimethylsiloxane (PDMS) as the structural material; and this has several drawbacks. Cyclo-olefin polymers (COPs) are more advantageous than PDMS and other thermoplastic materials because of their low drug absorption and autofluorescence. However, most COP-based microfluidic devices are fabricated by solvent bonding of the constituent parts. Notably, the remnant solvent can affect the cultured cells. This study employed a photobonding process with vacuum ultraviolet (VUV) light to fabricate microfluidic devices without using any solvent and compared their performance with that of solvent-bonded systems (using cyclohexane, dichloromethane, or toluene as the solvent) to investigate the effects of residual solvent on cell cultures. Quantitative immunofluorescence assays indicated that the coating efficiencies of extracellular matrix proteins (e.g., Matrigel and collagen I) were lower in solvent-bonded COP devices than those in VUV-bonded devices. Furthermore, the cytotoxicity of the systems was evaluated using SH-SY5Y neuroblastoma cells, and increased apoptosis was observed in the solvent-processed devices. These results provide insights into the effects of solvents used during the fabrication of microfluidic devices and can help prevent undesirable reactions and establish good manufacturing practices.

## 1. Introduction

Microfluidic technology is used for a variety of biological, industrial, and clinical applications, such as genomic and proteomic studies [1,2], in vitro diagnostics [3,4], and microphysiological systems (MPSs) or “organs-on-a-chip” [5,6,7,8,9]. MPSs hold great promise for the advancement of drug screening and toxicological testing because they can be used to predict the efficacy or toxicity of drugs without the need for animal experiments. With respect to drug screening and toxicology, ethical concerns have resulted in the transition from the use of animal models to other technologies [10], and MPSs containing human cells are an effective means of achieving this goal.

Many microfluidic MPSs employ polydimethylsiloxane (PDMS) or other polymers as their structural components. PDMS has good biocompatibility, gas permeability, optical transparency, low-cost, and ease of preparation. However, it has been reported that it may absorb hydrophobic molecules, leach uncrosslinked monomers, and enable water evaporation from the MPS [11,12,13,14,15], which might interfere with the cellular behavior and drug responses when used for microfluidic cell culture or drug toxicology. However, other researchers have reported that the absorption of PDMS is not problematic [16]. Still, PDMS presents other complications; the soft lithography processes used to manufacture PDMS-based MPSs are not suitable for mass production, because the elasticity of the PDMS microfluidic structures renders them difficult to handle [17], and PDMS-based MPSs cannot be stored for long durations because PDMS undergoes gradual crosslinking, resulting in structural shrinkage [13]. Alternatives to PDMS include glass [18,19] and silicon [20], which have long been used as structural materials for microfluidic devices. However, the fabrication of glass- and silicon-based MPSs often requires harsh reagents; glass-based microfluidic devices are very fragile and not suitable for mass production; and silicon-based microfluidic devices often use photoresist materials, which might have unexpected effects on cultured cells [21]. Therefore, polymers, such as polystyrene (PS), polymethyl methacrylate (PMMA), polypropylene (PP), and cyclo-olefin polymer (COP) are promising alternatives to PDMS for general cell cultures [15,17,22,23,24,25]. These materials can be produced on a large scale by simple molding processes, a promising strategy for the commercialization of MPSs [24,26].

Many polymers have certain limitations in terms of their optical properties [27]. For instance, PS and PMMA are known to autofluorescence, which interferes with the observation of cells by fluorescence microscopy. In addition, PP is not as transparent as other polymers to UV light, which is often used to excite fluorescence probes. In contrast, COP shows relatively low autofluorescence and good optical transparency [27], which would be beneficial for observing cells cultured in COP-based microfluidic devices. Therefore, COP is expected to be a promising material for the fabrication of microfluidic devices.

Bonding processes are required for assembling microfluidic devices made of two or more components. This can be achieved by using double-sided tape, glues, or solvent bonding, since the simple thermal bonding processes often are not sufficient to assemble [27]. Solvent bonding is the most practical of these techniques owing to its relative simplicity. However, the tape, glue, or solvent used for bonding can have an undesirable effect on cell culture and other assays owing to leakage issues and microstructural disruptions. Therefore, to reduce these effects, solvents are removed after solvent bonding by a vacuum process; however, small amounts of residual solvent remain, which can affect the cultured cells. Therefore, an alternative bonding process that does not require additional materials such as solvents is required to overcome these drawbacks. 

Photobonding using vacuum ultraviolet (VUV) light from an excimer lamp is a promising way of achieving this. Unlike glue- and solvent-based bonding processes, photobonding does not disrupt the microfluidic structures. Previously, we reported that VUV photobonding could be used to fabricate COP-based cell culture devices, resulting in reduced apoptosis of cultured human-induced pluripotent stem cells compared to that in solvent-bonded COP devices, which affected the cultured cellular phenotypes [28]. Thus, VUV photobonding could be beneficial for producing COP devices for cell culture and assays. However, the underlying mechanisms are not clearly understood.

In this study, we investigated the effect of residual solvents on the results of cell cultures and assays. Three typical solvents were used, toluene, cyclohexane, and dichloromethane. COP devices fabricated with VUV photobonding (COP-V devices) were used for comparison. The effects of these MPSs on extracellular matrix (ECM) protein coatings, which are generally used to facilitate cell adhesion and growth, were evaluated. Finally, SH-SY5Y cells, which are widely used to assess the cytotoxicity of chemicals and materials [29,30], were used to evaluate the cytotoxic effects of the solvent-processed COP devices. This investigation provides insights into the undesirable effects of solvent-processed COP devices on cell cultures, and is expected to aid the development of devices for precise cell cultures and assays.

## 2. Materials and Methods

### 2.1. Fabrication of COP-Based Microfluidic Structures and Plates

COP-based microfluidic devices were produced from two COP layers, i.e., the microfluidic structure and base plate. The fabricated COP devices consisted of eight microfluidic cell culture channels, each with a width, length, and depth of 800, 7500, and 250 µm, respectively [28]. The center-to-center distance between the channels was 9 mm. Each channel had a medium inlet and outlet (2 and 1 mm in diameter, respectively) and a total volume capacity of 13.2 µL [28].

A metal-molding process was used to fabricate the COP microfluidic structures and base plates, as described previously [28] (Figure S1 in Supplementary Materials). Briefly, the metal-molding process involved the use of metal molds to fabricate COP structures. COP (Zeonex 480R, Zeon, Tokyo, Japan) was injected into the molds, and then removed from the molds to obtain the COP microfluidic structures and base plates. Because VUV photobonding requires a greater hot-pressing load for successful bonding than the solvent-bonding process, COP microfluidic structures with depths of 290 and 320 µm (denoted as RC2 and RC3, respectively) were prepared for the solvent-bonded and VUV-photobonded devices, respectively.

### 2.2. Solvent-Bonding Process for COP Device Fabrication

**Figure 1:**
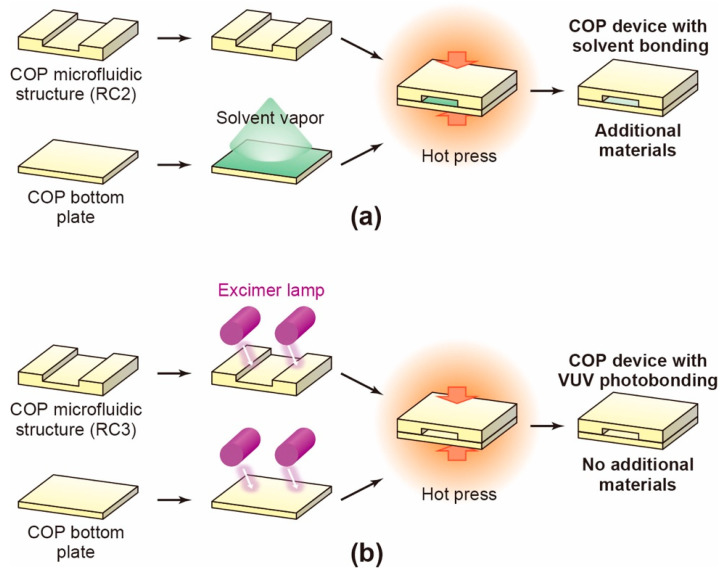
Schematic of the cyclo-olefin polymer (COP)-based microfluidic device fabrication process. Devices were prepared by (**a**) solvent bonding and (**b**) vacuum ultraviolet (VUV) photobonding. A solvent vapor or VUV light from an excimer lamp was used to bond the two components (microfluidic structure and base plate), which were then hot-pressed together to form the microfluidic device.

Solvent-bonding was conducted using three different solvents, namely, toluene, cyclohexane, and dichloromethane. The solvents were procured from Fujifilm Wako Pure Chemical Corporation (Osaka, Japan). As shown in Figure 1a, the base plate was exposed to the solvent vapor, and then the solvent-exposed base plate and unexposed microfluidic layer (RC2) were assembled and bonded by hot pressing under the conditions listed in Table 1. After this bonding process, the remaining solvent was evaporated in a constant-temperature bath at 60 °C for 24 h.

### 2.3. VUV-Photobonding Process for COP Device Fabrication

For the VUV-photobonding process, the COP microfluidic structure (RC3) and base plate were irradiated with VUV light (172 nm) from an excimer lamp (Ushio INC., Tokyo, Japan) at 25 °C. The surfaces of the components were then assembled and bonded by hot-pressing at 130 °C (Figure 1b).

### 2.4. Peeling Test

Peeling tests were conducted using a ZTS-50N digital force gauge (Imada, Aichi, Japan) (Figure S2 in Supplementary Materials) on bonded COP plates. COP plates with dimensions of 25 × 35 × 1 mm were overlapped by 8 mm along the short edge of the plates and joined by VUV photobonding (Section 2.3) or solvent bonding (Section 2.2). Thus, the bonded area was 25 × 8 mm. The bonded COP plates were placed on the stage of the force gauge (with a hollow space below the specimen), and the center of the bonded area was pressed at a rate of 20 mm/min until the sample disassembled or broke. 

### 2.5. Leakage Test

Leakage tests were performed using a 0.05% (*w*/*v*) solution of Acid Red 52 (Tokyo Chemical Industry, Tokyo, Japan) in phosphate-buffered saline (PBS) to visualize the leakage. Typically, 12 µL of the Acid Red 52 solution was introduced into each microfluidic channel of the COP devices and incubated at 37 °C in a humidified incubator. Then, the microfluidic channels were observed using a BX51 fluorescence microscope (Olympus, Tokyo, Japan) equipped with a MPLanFL N 5×/0.15 NA objective lens (Olympus), U-DM-CY3-3 filter cube (Olympus), U-LH100-3 lamp (Olympus), and DP21 digital camera (Olympus).

### 2.6. ECM Coating of Microfluidic Channels 

To produce ECM coatings on the COP devices, 15 µL of 0.26, 0.52, or 5.15 mg/mL Matrigel (Sigma-Aldrich, St. Louis, MO, USA), or 0.005%, 0.01%, or 0.1% (*w*/*v*) bovine collagen type I (Sigma-Aldrich) prepared in Dulbecco’s Modified Eagle medium (DMEM)/F12 (Sigma-Aldrich) was added to each microfluidic channel and incubated at 4 °C for more than 24 h. Excess Matrigel or collagen type I was removed, then the channels were washed with PBS.

### 2.7. Evaluation of the Amount of Coated ECM in the COP Devices

Immunostaining was used to quantify the amount of the coated Matrigel or collagen type I in the COP microfluidic channels. The ECM-coated microfluidic channels were fixed with 4% (*v*/*v*) paraformaldehyde prepared in PBS for 20 min at 25 °C, and then washed with PBS. Next, the ECM-coated channels were exposed to a blocking solution consisting of 5% (*v*/*v*) normal goat serum, 5% (*v*/*v*) normal donkey serum, 3% (*v*/*v*) bovine serum albumin (BSA), and 0.1% (*v*/*v*) Tween-20 prepared in PBS at 4 °C for 16 h. Then, the channels were incubated at 4 °C for 16 h with the following primary antibodies: anti-mouse laminin MEC5 (rat IgG; 1:50; Catalog number, ab2466; Abcam, Cambridge, UK), anti-mouse collagen IV (rabbit IgG; 1:100; Catalog number, ab19808; Abcam), anti-mouse entactin (NID 1; 1:50; mouse IgG; Catalog number, MBS603630; MyBioSource, San Diego, CA, USA), or anti-rabbit collagen I (rabbit IgG; 1:50; Catalog number, ab34710; Abcam) in prepared in blocking solution at 4 °C for 16 h. The channels were subsequently incubated at 25 °C for 60 min with the corresponding secondary antibodies, Alexa Fluor 594 donkey anti-rabbit IgG (1:1000; Jackson ImmunoResearch, West Grove, PA, USA), and Alexa Fluor 648 donkey anti-rat IgG (1:1000; Jackson ImmunoResearch) prepared in blocking solution. The stained samples were placed on the imaging stage of a Nikon ECLIPSE Ti inverted fluorescence microscope equipped with a CFI Plan Fluor 10×/0.30 NA objective lens (Nikon, Tokyo, Japan), charge-coupled device (CCD) camera (ORCA-R2; Hamamatsu Photonics, Hamamatsu City, Japan), mercury lamp (Intensilight; Nikon), XYZ automated stage (Ti-S-ER motorized stage with encoders; Nikon), and filter cubes for the fluorescence channel (TRITC and CY5 HYQ; Nikon). Once the microscopy images were acquired, CellProfiler software (Broad Institute of Harvard and MIT, Version 3.1.9) was used to quantify the stained ECMs. Box plots were generated using R software (ver. 3.5.2; https://www.r-project.org/, accessed on 20 December 2018).

### 2.8. Cell Culture

SH-SY5Y human neuroblastoma cells were obtained from the American Type Culture Collection. The cells were maintained in DMEM (Sigma-Aldrich) supplemented with 10% (*v*/*v*) fetal bovine serum (FBS, SAFC Bioscience, Lenexa, KS, USA), 2 mM l-glutamine (200 mM, Thermo Fisher Scientific, Inc., Waltham, MA, USA), and 20 mM HEPES (Fujifilm Wako) in a humidified incubator at 37 °C with 5% (*v*/*v*) CO_2_. The cells were flushed with trypsin/EDTA (0.04%/0.03% [*v*/*v*]) solution every four days at a ratio of 1:4 (*v*/*v*).

### 2.9. Cell Culture in the COP Devices

Before use, the COP microfluidic devices were wiped with 70% (*v*/*v*) EtOH and placed under UV light in a biosafety cabinet for 30 min. Then, as described in Section 2.5, 10 µL of the ECM solution prepared in DMEM/F12 was introduced into each microfluidic cell culture channel and incubated at 4 °C for >24 h. Subsequently, the excess ECM solution was removed, and the coated channels were washed with fresh medium.

SH-SY5Y cells cultured in a dish were harvested with 1 mL of TrypLE Express at 37 °C for 5 min and transferred to a 15-mL centrifuge tube. Thereafter, 4 mL of the cell culture medium was added to the tube, and the cells were centrifuged at 200× *g* for 3 min. Once the supernatant was decanted, the cells were resuspended in 5 mL medium and centrifuged at 200× *g* for 3 min. Next, the cells were resuspended in the medium at 2.0 × 10^6^ cells per 10 µL and introduced into the COP microfluidic channels. Two hours after seeding, fresh medium was added to the microfluidic cell culture channels to remove debris. The medium was changed every 12 h until further experiments were performed.

### 2.10. Apoptosis Assays

Annexin V staining was performed according to the manufacturer’s instructions for the Alexa Fluor 594-Annexin V conjugate (Molecular Probes, Eugene, OR, USA). Briefly, the cells were washed with annexin-binding buffer (10 mM HEPES, pH 7.4, 140 mM NaCl, 2.5 mM CaCl_2_) and then stained with Alexa Fluor 594-Annexin V conjugate at 25 °C for 15 min. Following fixation with 4% (*v*/*v*) paraformaldehyde prepared in PBS at 25 °C for 15 min, the cells were incubated with 300 nM of Hoechst 33258 at 25 °C for 30 min.

### 2.11. Microscopic Cell Imaging

Samples containing cells were placed on the imaging stage of a Nikon ECLIPSE Ti inverted fluorescence microscope equipped with a CFI Plan Fluor 10×/0.30 NA objective lens (Nikon), CCD camera (ORCA-R2; Hamamatsu Photonics), mercury lamp (Intensilight; Nikon), XYZ automated stage (Ti-S-ER motorized stage with encoders; Nikon), and filter cubes for the fluorescence channels (DAPI, GFP HYQ, and TRITC; Nikon).

### 2.12. Single Cell Profiling Based on Microscopic Images

The cells in the microscopy images were identified using CellProfiler software (Broad Institute of Harvard and MIT, Version 3.1.9) via Otsu’s method [31]. The fluorescence signals from individual cells were quantified automatically. Box plots were generated using R software (ver. 3.5.2; https://www.r-project.org/, accessed on 20 December 2018).

### 2.13. Statistical Analysis

The *p* values were estimated using Steel–Dwass and Wilcoxon signed rank tests and R software (ver. 3.5.2; https://www.r-project.org/, accessed on 20 December 2018).

## 3. Results

### 3.1. Fabrication of COP-Based Microfluidic Devices

To evaluate the effects of the solvent used in the solvent-bonding process, we prepared COP microfluidic devices with three commonly used solvents, namely, toluene [32], cyclohexane [33,34], and dichloromethane [35]. The devices were denoted as COP-T, COP-C, and COP-D, respectively (Figure 1a and Table 1). For comparison, a COP microfluidic device was fabricated via a VUV-photobonding process without using any solvent (Figure 1b); this device was denoted as COP-V [28]. We also prepared a thermally bonded COP device without solvent bonding or VUV photobonding, but the device was not fabricated well and exhibited leakage, cracking, and disassembly during the cell culture experiments. Therefore, we could not use the thermally bonded COP device as a control sample. 

The target height of the microfluidic channels was 250 μm. However, the channel height is reduced during the hot-pressing process. Therefore, the COP microfluidic structures were designed with deeper channels than the target height. Because VUV photobonding requires a greater hot-pressing load for successful bonding than that required in the solvent-bonding process, COP microfluidic structures were prepared with heights of 290 and 320 μm (denoted as RC2 and RC3, respectively; Figure S3 in Supplementary Materials) for use in the solvent-bonded and VUV-photobonded devices, respectively (Table 2).

**Figure 2:**
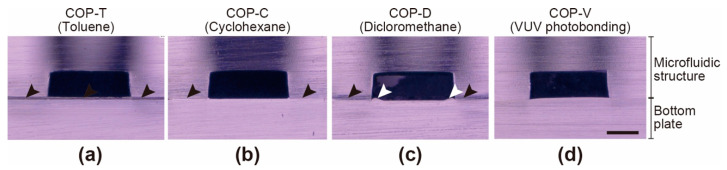
Cross-sections of microfluidic channels in cyclo-olefin polymer (COP)-based microfluidic devices. The devices were prepared by assembling a microfluidic structure and base plate by solvent bonding using (**a**) toluene (COP-T), (**b**) cyclohexane (COP-C), and (**c**) dichloromethane (COP-D); and (**d**) by a vacuum ultraviolet (VUV)-photobonding process without any solvent (COP-V). The white arrows indicate deformation of the microfluidic channel. The black arrows indicate the color change in the COP material at the bonding interface. Scale bar: 300 µm.

Solvent exposure can result in the deformation of polymers such as COP. To minimize structural deformation, the solvent-bonding processes were carefully optimized for each solvent (Table 1). Figure 2 shows cross-sectional micrographs of the microfluidic channels in the COP devices. The COP-T, COP-C, and COP-V devices did not show any noticeable deformation; however, deformation of the microfluidic channels was observed in the COP-D device after hot pressing (white arrows in Figure 2c). Moreover, in the COP-T, COP-C, and COP-D devices, the interface between the two COP components exhibited color changes, as shown by the black arrows in Figure 2. This suggests that solvent bonding alters the surface properties of the COP, which might affect cell culture. In contrast, the COP-V device did not show any color variation. Moreover, the interface in the COP-V device could not be observed clearly, because the components were tightly assembled with no pronounced changes in appearance.

**Figure 3:**
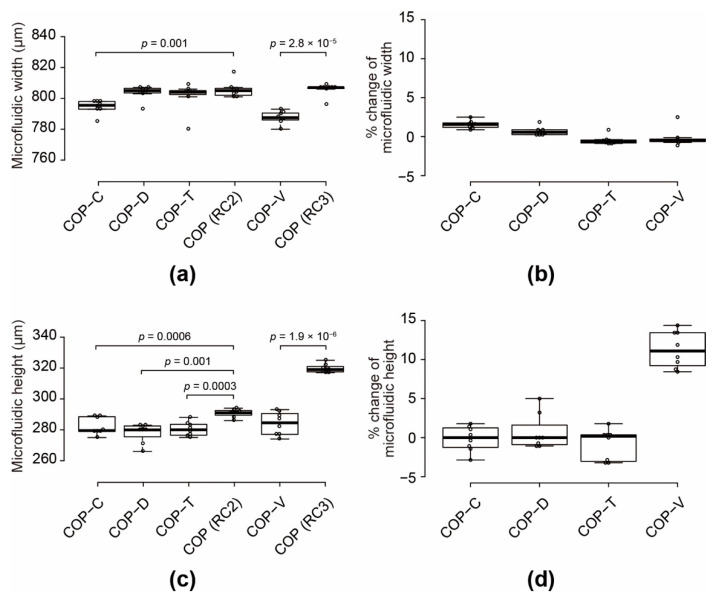
Variations in the width and height of cyclo-olefin polymer (COP) microfluidic channels: (**a**,**c**) measured widths and heights, respectively, and (**b**,**d**) deviations of the widths and heights, respectively, relative to those of the original structures. The center lines indicate the median values; the box limits indicate the 25th and 75th percentiles; the whiskers extend to 1.5-times the interquartile range from the 25th and 75th percentiles; outliers are represented by dots; and data points are plotted as open circles. The devices were processed by solvent bonding with toluene (COP-T), cyclohexane (COP-C), or dichloromethane (COP-D); or by vacuum ultraviolet (VUV) photobonding (COP-V). RC2 and RC3 are the original structures for the solvent-bonding and VUV-photobonding processes, respectively. Eight samples of each type were analyzed.

The deformation of the COP microfluidic structures and devices after bonding and hot pressing was evaluated quantitatively (Figure 3). The widths and heights of the COP microfluidic structures (RC2 and RC3) had a coefficient of variation (CV) of less than 1% of the original values after removal from the metal molds. The widths of the COP-C and COP-V specimens decreased by 1.4 and 2.2%, respectively, compared to those of the original structures (Figure 3a,b). The other specimens showed width reductions of less than 1%. The solvent-bonded microfluidic devices showed height reductions of approximately 3–5% with a CV of 2.5%. In contrast, the height of the COP-V specimen decreased by 11.2% relative to that of the original structure owing to the greater hot-pressing load; therefore, the final height was close to the target height of 250 μm (Figure 3c,d). These results indicate that the solvent-bonding processes did not cause noticeable deformation of the microfluidic structures. For a reasonable comparison of the effects of the solvents on the cultured cells, it is necessary to eliminate all other factors.

**Figure 4:**
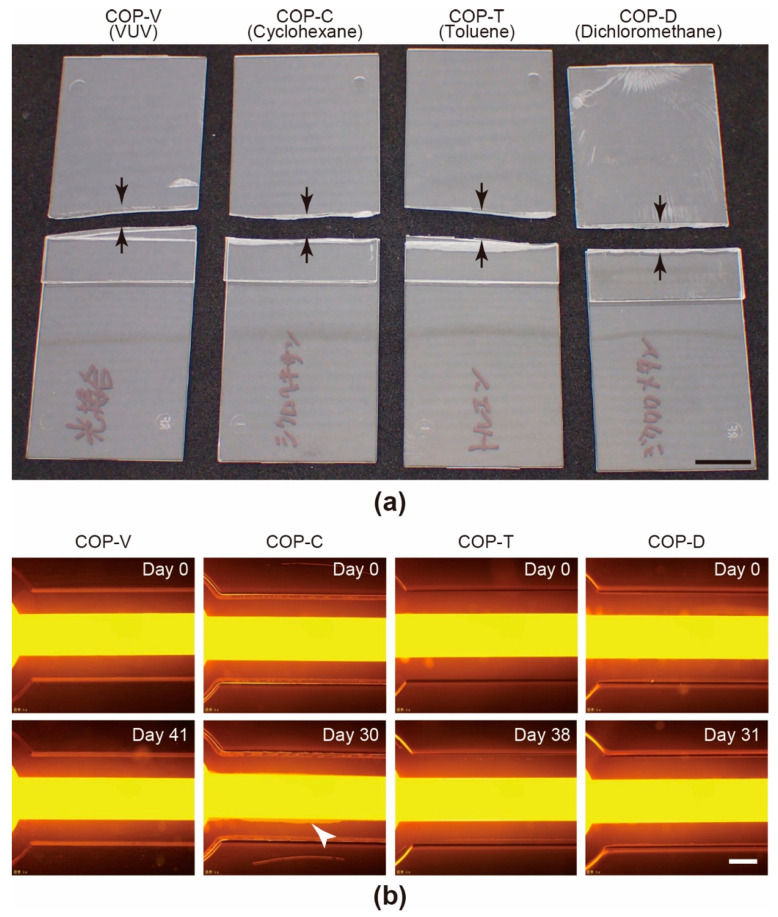
(**a**) Photographs captured after the peeling tests conducted to evaluate the bonding strengths of cyclo-olefin polymer (COP) microfluidic devices fabricated by vacuum ultraviolet (VUV) photobonding (COP-V) or solvent bonding using cyclohexane (COP-C), toluene (COP-T), or dichloromethane (COP-D). Scale bars: 10 mm. (**b**) Fluorescent micrographs captured after leakage tests of COP microfluidic channels fabricated by solvent- and VUV-bonding processes. To visualize the leakage, a 0.05% solution of Acid Red 52 was introduced into the MPS microfluidic channels and incubated. The white arrowhead indicates the leakage area for the COP-C device. Scale bars: 500 µm.

To evaluate the adhesion strength of the COP components assembled by different bonding processes, peeling tests were carried out on bonded COP plates (Figure 4a). All the tested bonding conditions facilitated strong assembly of the COP plates, and the bonded plates could not be disassembled without breaking the plates.

Leakage tests were conducted to evaluate the sealing of the COP microfluidic devices prepared by different bonding processes. We introduced a solution of fluorescent dye, Acid Red 52, into the microfluidic channels and incubated the devices under conditions similar to those used for cell culture (Figure 4b). While the COP-C device showed a small amount of liquid leakage after 30 days of incubation, as indicated by the white arrowhead in Figure 4b, the COP-V, COP-T, and COP-D devices did not show any leakage even after 31–41 days of incubation.

### 3.2. Effect of Residual Solvent on the ECM Coating in COP-Based Microfluidic Devices

There are concerns that trace amounts of solvent could affect the ECM-coating process or disrupt ECM proteins. Although it is difficult to distinguish between the causes of coating process or disruption, the effect of solvent residues could be evaluated by studying the coated ECMs. To evaluate this, we immunostained ECM proteins coated on the surface of the COP microfluidic channels. We used Matrigel and collagen type I (Col I) as ECMs, which are used in a variety of cell cultures, and stained them with specific antibodies for their quantitative analysis. 

**Figure 5:**
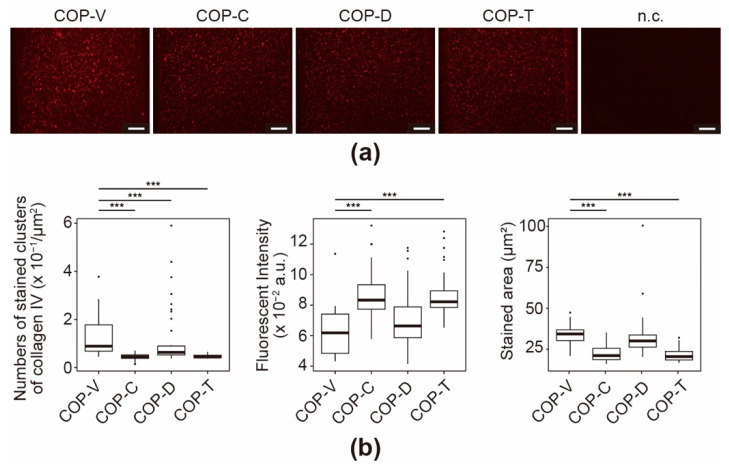
Immunofluorescence for collagen IV (Col IV) in Matrigel-coated cyclo-olefin polymer (COP) microfluidic channels. (**a**) Micrographs of immunostained Col IV in Matrigel-coated COP microfluidic channels processed using vacuum ultraviolet (VUV) photobonding (COP-V) or solvent bonding with cyclohexane (COP-C), toluene (COP-T), or dichloromethane (COP-D). n.c. represents the negative control of a COP-V microfluidic channel coated with Matrigel and stained with a secondary antibody. Scale bars: 100 µm. (**b**) Quantitative analyses of the immunostained Col IV in the Matrigel-coated COP microfluidic channels. The center lines indicate the median values; the box limits indicate the 25th and 75th percentiles; the whiskers extend to 1.5-times the interquartile range from the 25th and 75th percentiles; and outliers are represented by dots. Each experiment was repeated four times, and more than ten images of the COP devices from each experiment were used for quantification. *** *p* < 0.005.

The major components of Matrigel are collagen type IV (Col IV), laminin, and entactin (also referred to as Nidogen-1) [36,37,38,39]; therefore, we performed immunostaining assays with each of the respective antibodies. In the case of Col IV, the COP devices with coated microfluidic channels showed higher fluorescence than the negative control (n.c.) (see Figure 5a). In particular, the COP-V devices had many stained clusters with strong fluorescence. These clusters correspond to fibrous collagen structures, which are required for the optimal functioning of the ECM. Owing to the nature of such collagen fiber structures under in vivo physiological conditions, the stained clusters are a good indication of the coating efficiency. In contrast, the COP-T, COP-C, and COP-D devices showed fewer immunostained clusters, suggesting that the residual solvents affected the coating process or disrupted the Matrigel coating. The measured fluorescence intensity had an inverse relationship with the stained area (Figure 5b). This suggests that the Col IV proteins attached to the surface of COP-V microfluidic channels formed larger and flatter clusters than those in the other devices, leading to decreased fluorescence intensity. Although the total fluorescence intensities of the stained clusters in the COP-C and COP-T devices were higher than those in the COP-V and COP-D devices, the number of stained clusters and the stained areas in COP-C and COP-T devices were much lower than those in the COP-V and COP-D devices. These results suggest that cyclohexane and toluene affected the coating of Col IV on the surface of the COP microfluidic channel.

**Figure 6:**
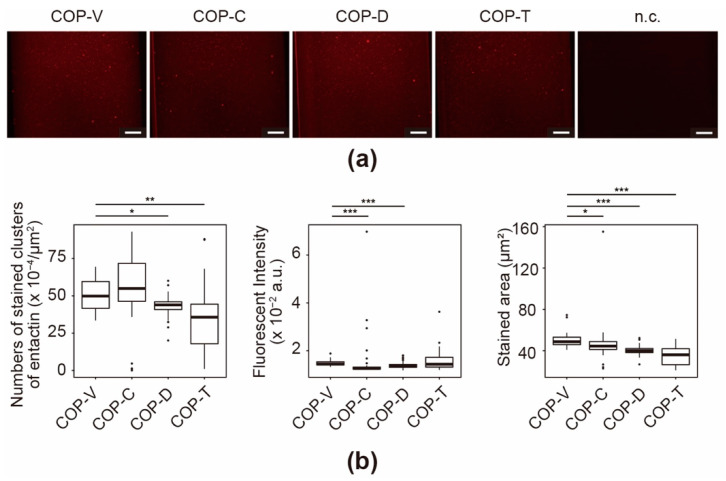
Immunofluorescence for entactin in Matrigel-coated cyclo-olefin polymer (COP) microfluidic channels. (**a**) Micrographs of immunostained entactin in Matrigel-coated COP microfluidic channels processed using vacuum ultraviolet (VUV) photobonding (COP-V) or solvent bonding with cyclohexane (COP-C), toluene (COP-T), or dichloromethane (COP-D). n.c. represents the negative control of a COP-V microfluidic channel coated with Matrigel and stained with a secondary antibody. Scale bars: 100 µm. (**b**) Quantitative analyses of the immunostained entactin in the Matrigel-coated COP microfluidic channels. The center lines indicate the median values; the box limits indicate the 25th and 75th percentiles; the whiskers extend to 1.5-times the interquartile range from the 25th and 75th percentiles; and outliers are represented by dots. Each experiment was repeated four times, and more than ten images of the COP devices from each experiment were used for quantification. * *p* < 0.05, ** *p* < 0.01, *** *p* < 0.005.

The immunoassays for entactin revealed increased florescence of the stained microfluidic channels in all the stained COP devices compared with that observed in the negative control (n.c.) (Figure 6a). In particular, the COP-V and COP-D devices showed stronger fluorescence. However, unlike in case of Col IV, few stained clusters were observed. Although entactin has a domain that can bind to Col IV to form a basement membrane in vivo [39], it showed fewer Col IV-like clusters, and covered the entirety of the microfluidic channels under in vitro conditions (Figure 6b). This is because the entactin protein itself does not have the ability to form fibrous structures like collagen. The fluorescence microscopic images in Figure 6a reveal that cyclohexane and toluene affected the entactin coating.

**Figure 7:**
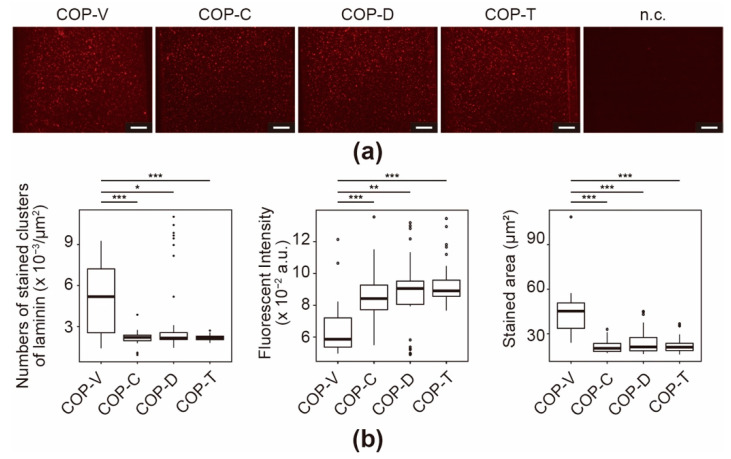
Immunofluorescence for laminin in Matrigel-coated cyclo-olefin polymer (COP) microfluidic channels. (**a**) Micrographs of immunostained laminin in Matrigel-coated COP microfluidic channels processed using vacuum ultraviolet (VUV)-photobonding (COP-V) or solvent-bonding with cyclohexane (COP-C), toluene (COP-T), or dichloromethane (COP-D). n.c. represents the negative control of a COP-V microfluidic channel coated with Matrigel, and only stained with a secondary antibody. The scale bars represent 100 µm. (**b**) Quantitative analyses of immunostained laminin in the Matrigel-coated COP microfluidic channels. The center lines represent the median values; the box limits indicate the 25th and 75th percentiles; the whiskers extend to 1.5-times the interquartile range from the 25th and 75th percentiles; and outliers are represented by dots. Each experiment was repeated four times, and more than ten images of the COP devices from each experiment were used for quantification. * *p* < 0.05, ** *p* < 0.01, *** *p* < 0.005.

In the laminin immunofluorescence assays, fluorescence signals were observed in the Matrigel-coated COP microfluidic channels (Figure 7a), and stained clusters were also observed (Figure 7b). The COP-V devices showed significantly more clusters than the solvent-bonded devices. Moreover, similar to that for Col IV, the fluorescence intensity showed an inverse correlation with the stained area. Laminin does not form fibrous structures, but it interacts strongly with Col IV; hence, fluorescent clusters were observed. As shown in Figure 7b, the solvent used did not affect the number, fluorescence intensity, or stained area of the clusters, but the results of the solvent-bonded devices were significantly different from those of the COP-V devices. In summary, the solvents have different effects on each component of Matrigel, and thus might affect the result of cell culture assays.

**Figure 8:**
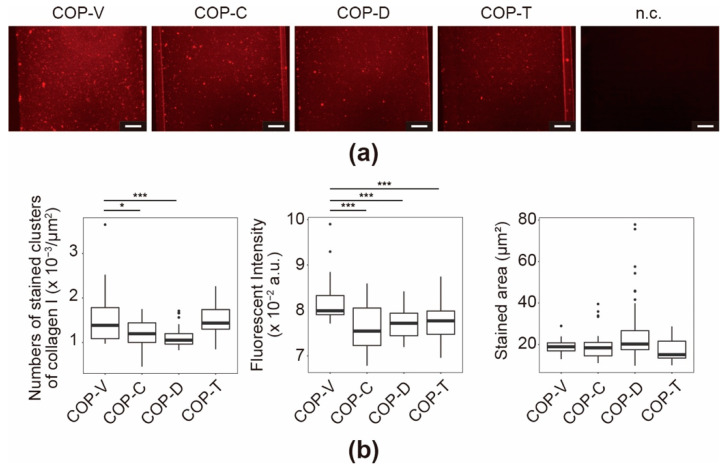
Immunofluorescence for collagen I (Col I) in Col I-coated cyclo-olefin polymer (COP) microfluidic channels. (**a**) Micrographs of immunostained Col I in Col I-coated COP microfluidic channels processed using vacuum ultraviolet (VUV)-photobonding (COP-V) or solvent-bonding with cyclohexane (COP-C), toluene (COP-T), or dichloromethane (COP-D). n.c. represents the negative control of a COP-V microfluidic channel coated with Col I and stained with a secondary antibody. Scale bars: 100 µm. (**b**) Quantitative analyses of immunostained Col I in the Col I-coated COP microfluidic channels. The center lines represent the medians; the box limits indicate the 25th and 75th percentiles; the whiskers extend to 1.5-times the interquartile range from the 25th and 75th percentiles; and outliers are represented by dots. Each experiment was repeated four times, and more than ten images of the COP devices from each experiment were used for quantification. * *p* < 0.05, *** *p* < 0.005.

The immunostaining results of the COP devices coated with Col I were similar to those obtained for Col IV in the Matrigel-coated devices (Figure 8a). The COP-V devices showed widely distributed fluorescence signals across the microfluidic channels, with many fluorescent stained clusters of Col I fibers. As mentioned above, because collagen fibers are required to express the collagen functionality, the presence of Col I clusters is a good indicator of the coating efficiency. In comparison, the COP-T, COP-C, and COP-D devices showed fewer immunostained clusters. Quantitative analysis was performed based on the fluorescence micrographs to evaluate the number of stained clusters, the fluorescence intensities, and the stained area (Figure 8b). There were fewer stained clusters in the COP-C and COP-D devices than in the COP-V and COP-T devices. In addition, the stained clusters in the COP-V devices exhibited the strongest fluorescence intensities. Interestingly, the COP-V devices showed the least variation in stained area, while the COP-D devices showed the most variation. These results suggest that the solvents altered the surface properties of the COP device and interfered with the efficacy of the Col I coating.

### 3.3. Effect of Solvent on SH-SY5Y Cells Cultured in COP Devices

**Figure 9:**
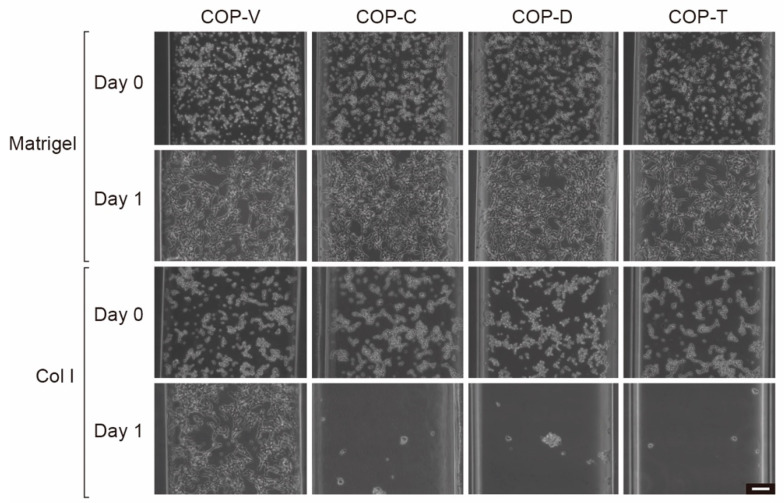
Micrographs of SH-SY5Y neuroblastoma cells cultured in cyclo-olefin polymer (COP) microfluidic devices fabricated using vacuum ultraviolet (VUV)-photobonding (COP-V) or solvent-bonding with cyclohexane (COP-C), toluene (COP-T), or dichloromethane (COP-D). The COP microfluidic channels were coated with Matrigel or collagen I (Col I). Scale bar: 100 µm.

SH-SY5Y neuroblastoma cells were used to evaluate the cell culturing behavior of the devices (Figure 9). Immediately after the cells were loaded into the microfluidic channels of the COP devices (day 0), the SH-SY5Y cells in the Matrigel-coated COP devices showed a flattened cellular shape. In contrast, the cells in the Col I-coated COP devices showed a circular cellular shape. These results suggest that the cells in the Matrigel-coated COP devices adhered to the substrate more efficiently. Moreover, one day after cell loading, the cells in the Matrigel-coated COP devices had adhered and spread, whereas the cells in the Col I-coated COP devices, except for those in the Col I-coated COP-V device, were removed by the medium when it was changed. As shown in Figure 8, the microfluidic channels in the COP devices could be coated with Col I, but the residual solvent affected the coating efficiency. Consequently, the cells cultured in the Col I-coated COP-C, COP-D, and COP-T devices were not viable, even for one day. This result suggests that residual solvent might directly affect not only the Col I-coating efficiency, but also cell adhesion or survival. In contrast, the cells remained viable in the Matrigel-coated devices, suggesting that the entactin and laminin in Matrigel cooperated to support cell adhesion to the Col IV proteins and cell survival. Therefore, only the Matrigel-coated COP devices were used in subsequent evaluations.

**Figure 10:**
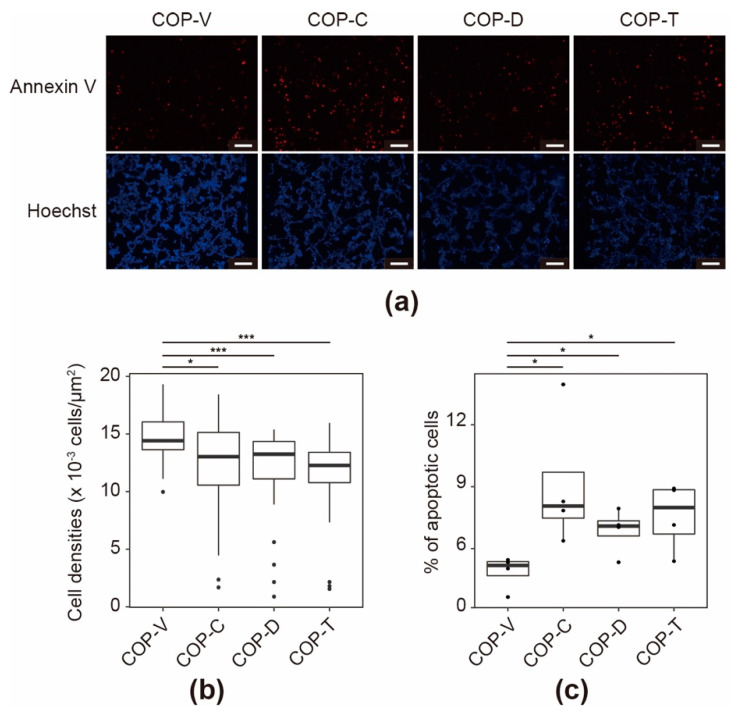
Evaluation of apoptotic cells cultured in cyclo-olefin polymer (COP) devices prepared by vacuum ultraviolet (VUV)-photobonding (COP-V) or solvent-bonding with cyclohexane (COP-C), toluene (COP-T), or dichloromethane (COP-D). (**a**) Fluorescence micrographs of SH-SY5Y neuroblastoma cells stained with Annexin V-Alexa 594 apoptotic marker. SH-SY5Y cells were cultured in Matrigel-coated COP devices for four days and then stained with fluorescent dyes. Hoechst 33258 (Hoechst) was used as the fluorescent dye to visualize the cell nuclei. Scale bars: 100 µm. (**b**) Cellular densities after four days of culturing in the COP devices, as determined by counting the nuclei. (**c**) Percentage of apoptotic cells after four days of culturing in the COP devices, as determined from the Annexin V-Alexa 594 positive cells (Figure S4 in the Supplementary Materials). The center lines represent the median values; the box limits indicate the 25th and 75th percentiles; the whiskers extend to 1.5-times the interquartile range from the 25th and 75th percentiles; and outliers are represented by dots. Each experiment was repeated four times, and more than ten images of the COP devices from each experiment were used for quantification. * *p* < 0.05, *** *p* < 0.005.

After four days of culturing in the COP devices, the cell populations and apoptotic status of the SH-SY5Y cells were evaluated by staining them with Hoechst 33258 fluorescent dye and an Annexin V apoptotic marker labeled with Alexa 594 fluorescent dye (Annexin V-Alexa 594) (Figure 10a). This was followed by quantitative single-cell profiling of the fluorescence intensity of each cell to estimate the cell densities (Figure 10b) and apoptotic cell numbers (Figure 10c and Figure S4 in Supplementary Materials). The cell densities in the COP-V devices were significantly higher than those in the COP-C, COP-D, and COP-T devices (*p* = 2.4 × 10^−2^, 5.1 × 10^−4^, and 7.4 × 10^−6^, respectively, as estimated by Wilcoxon signed rank tests). In the apoptosis analysis, the micrographs of the cells stained with Annexin V-Alexa 594 indicated that there were more positive cells in the COP-C and COP-T devices than there were in the COP-V and COP-D devices. Apoptotic cells were counted by measuring the fluorescence intensity of the Annexin V-Alexa 594 dye. When the fluorescence intensity of the stained cells exceeded 0.1-times the fluorescence intensity of the Annexin V-Alexa 594 dye, they were defined as apoptosis “positive” cells (Figure 10c). Cells cultured in COP-V devices showed 4.9 ± 0.7% of apoptotic cells, whereas those cultured in COP-C, COP-D, and COP-T devices showed significantly higher percentages of apoptotic cells (9.1 ± 2.9, 6.7 ± 0.9, and 7.5 ± 1.4%, respectively; *p* < 0.05), as estimated by Student’s *t*-tests. Therefore, the solvents not only disrupted the ECM coating, but also altered the cellular status by reducing the cell adhesion and inducing cellular apoptosis.

## 4. Discussion

Microfluidic technology is used in several applications. In particular, microfluidic MPSs and organs-on-a-chip hold great promise as alternatives to animal models for drug screening and toxicology testing. However, there are a number of issues that must be overcome before MPSs can be widely adopted. Owing to the issues related to the use of PDMS as the structural material, most studies on MPSs have focused on finding alternative materials [11,12,13,14]. Although other polymers, such as PS, PMMA, COP, and photoresists have been used for fabricating microfluidic devices [15,17,22,23,24], they might also alter the cellular behavior. For example, we previously found that microfabrication materials, including PDMS, induce changes in cellular phenotypes and gene expression [21]. The present study demonstrates that in addition to the structural materials of the microfluidic devices, the solvents used in the bonding process can also strongly influence the growth of cells cultured in the microfluidic devices. In fact, each thermoplastic material requires a different bonding process [40], and the effects of both the structural material and the residual solvent on the cultured cells should be investigated to prevent incorrect interpretation of the results.

This study presents an approach for assessing how the materials used in the microfluidic device fabrication process affect cell culture. We consider four key factors, i.e., (1) the microfluidic structure, (2) solvent residue, (3) ECMs, and (4) cell types, which have not yet been discussed in detail in the evaluation of microfluidic devices. To understand the effects of these factors, it is necessary to use chips with the same simple structure but composed of different materials and processed with different solvents to eliminate the effects of additional parameters. This is a major challenge with research related to microfluidic devices for cell culture. Although several microfluidic devices have been developed, their structures, materials, cell handling, and analyses are different. Therefore, researchers and users have difficulty in judging the materials and conditions that are best suited for the target application. Consequently, it is not possible to investigate and compare the effects of the material as well as the solvent on the cultured cells in different microfluidic devices. As in our previous study [28], this study used a simple and identical microfluidic structure with one inlet and outlet for each microfluidic channels, but used a different material. Further, the microfluidic devices were processed using different solvents for clear and reasonable comparison. This approach will help both researchers studying microfluidic devices and users to understand the characteristics of microfluidic devices in detail.

Quantitative immunostaining was performed to investigate whether residual solvent disrupted and/or inhibited the ECM-coating process. When cells are cultured in microfluidic devices, the first step is cell–ECM interaction; if this interaction does not occur properly, the cells cannot adhere, grow, or survive; nor can they be used for further assays. In this study, we tested Matrigel (a mixture of Col IV, entactin, and laminin) and Col I, which are commonly used ECMs for cell culture in MPSs. The results indicated that the amounts of ECMs in solvent-bonded microfluidic devices were markedly reduced compared to those in the VUV-photobonded devices. Owing to the fibrous structure of collagen—in addition to the fluorescence intensity—the number and area of fibrotic clusters can be used to investigate the ECM coating efficiency. Thus, we confirmed that the residual solvent reduced the amount of ECM coated on the microfluidic surfaces. It is important to note that the ratios of the components of the Matrigel ECM were also affected by the solvent. As ECM proteins trigger intracellular signaling pathways via corresponding ECM receptors (e.g., integrins) [41,42,43,44], the functioning of ECM-associated pathways would be altered by changes in the ECM ratios. Thus, it is necessary to understand the characteristics of ECM coatings for cell culture and assays.

Finally, SH-SY5Y neuroblastoma cells were used to investigate the cytotoxic effects of the residual solvents. Using a commonly used cell line for standardized cell-based toxicological assays [29,30], it was possible to compare the results with those of other platforms used for cell cultures and assays. In this study, cell adhesion and apoptosis caused by leaked solvents were evaluated using this cell line. All the tested solvents markedly decreased cell adhesion and increased the number of apoptotic cells. As mentioned earlier, the ECMs on the COP surface were disrupted by the leaked solvent. Therefore, the results obtained for the cells should be considered in combination with the ECM results.

This article demonstrates an approach for evaluating the effects of the solvents and material used for microfluidic device fabrication on cultured cells. Although only SH-SY5Y neuroblastoma cells were used in the study, the selection of cell type is important and depends on the target application. As each microfluidic device is fabricated for a specific purpose in terms of simulating tissue or organ function in vitro, it is necessary to select representative tissue cell lines to confirm whether they can demonstrate optimal functions in microfluidic devices. Therefore, the cellular function assays—used for evaluation—should be selected carefully. Pluripotent stem cells (PSCs) [45,46,47] are beneficial in this respect because they provide differentiating cells during early development and are very sensitive to changes in their environments [48,49]. PSCs also provide a collection of tissue cells from the same cell source, eliminating concerns regarding genomic differences among established cell lines. It is also possible to evaluate the genomic abnormalities and differences in sensitivity caused by solvents and materials among tissue cells derived from PSCs. Recent advances in a variety of “omics” technologies will provide deeper insights into the molecular mechanisms underlying the effects of MPS materials on cellular behaviors [50]. This information can minimize misleading results obtained with MPSs and improve the choice of materials and fabrication processes for synthesizing MPSs with minimal adverse effects.

Microfluidic technology can also be applied for in vitro cancer diagnosis with very small amounts of cancer tissue from patient biopsy specimens, and to predict the efficacy and side effects of anti-cancer drugs [3,51,52]. Although cancer cells seem to tolerate stressful environments, cancer specimens contain a mixture of cells, and the cellular population ratios would be critical for understanding the cancer and patient characteristics. As demonstrated, solvents and materials could alter the features of ECM and cultured cells, thereby resulting in the generation of misleading results during anti-cancer drug treatments.

Overall, this work presents a method for evaluating whether a solvent used for microfluidic device fabrication is appropriate and the effect of the residual solvent in the microfluidic device on the cultured cells. Despite numerous attempts to eliminate processing solvents from MPSs, their complete removal has remained a challenge. We demonstrate that solvent residues affect the ECM-coating process and disrupt the ECMs. We also show that SH-SY5Y neuroblastoma cells undergo apoptosis in solvent-treated microfluidic devices.

## 5. Conclusions

In this study, we investigated the effects of solvents used in the processing of microfluidic devices on cultured cells by using solvent-bonded and VUV-photobonded microfluidic devices. By comparing the characteristics of four identical COP-based microfluidic devices, we could reasonably assess the effects of different solvents on the status of cultured cells. Solvent-bonding processes can cause the deformation of the microfluidic structures, which can be minimized by optimizing the process for each solvent. Thus, concerns regarding inconsistencies between fabricated structures can be eliminated. The COP devices were used to study differences in cell cultures and to determine the changes in the efficiencies of extracellular matrices (ECMs) coated on the surface of COP microfluidic channels. The solvents were found to reduce the levels of ECMs attached to the channel surfaces. Finally, the effects on cell adhesion and apoptosis—fundamental aspects studied in cell cultures—were investigated. The results indicated that solvent residues influence all the tested cell behaviors, leading to non-optimal cell cultures and assays. Thus, MPSs fabricated via solvent bonding are not suitable for cell culture and assays, because cellular status could not be adequately observed during culture. Our approach of investigating the effects of the materials used in the MPS fabrication is an essential step before further application of MPSs in drug screening and toxicology. Moreover, it could be the first step toward a deeper understanding of the effects of the microfluidic device components and could aid the development of microfluidic devices with optimal cellular behaviors for advanced drug screening.

## Figures and Tables

**Table 1:** Parameters of the solvent-bonding processes.

Solvent	Cyclohexane	Dichloromethane	Toluene
Temperature	30 °C	30 °C	30 °C
Exposure time	3 min	1 min 30 s	4 min 30 s
Hot press strength	3 kN (1200 N/cm^2^)	1 kN (400 N/cm^2^)	1 kN (400 N/cm^2^)
Hot press time	5 min	5 min	5 min
Hot press temp.	90 °C	90 °C	90 °C

**Table 2:** Summary of cyclo-olefin polymer (COP) microfluidic structures, target heights, and bonding processes.

Fabrication Parameters	COP-V ^1^	COP-C ^2^	COP-D ^2^	COP-T ^2^
COP microfluidic structure	RC3	RC2	RC2	RC2
Channel height	320 µm	290 µm	290 µm	290 µm
Target height	250 µm	250 µm	250 µm	250 µm
Bonding process	Vacuum ultraviolet (VUV) photobonding	Solvent bonding (cyclohexane)	Solvent bonding (dichloromethane)	Solvent bonding (toluene)

^1^ COP device prepared by vacuum ultraviolet (VUV) photobonding (COP-V). ^2^ COP devices prepared by solvent bonding in cyclohexane (COP-C), dichloromethane (COP-D), and toluene (COP-T).

## Data Availability

The data presented in this study are available on request from the corresponding author.

## Supplementary Materials

The following materials are available online at https://www.mdpi.com/article/10.3390/mi12050550/s1, Figure S1: Metal-molding process for fabricating the microfluidic structure of COP devices, Figure S2: Experimental setup of the peeling test used to evaluate the bonding strength of the COP devices, Figure S3: Cross-sections of the RC2 and RC3 COP microfluidic structures, Figure S4: Histograms of the quantitative single-cell profiling of apoptotic cells stained with Annexin V labeled with Alexa 594.
