# Supplementary material for: Evaluation of the Effects of Solvents Used in the Fabrication of Microfluidic Devices on Cell Cultures
Source: Micromachines (Basel). 2021 May 12;12(5):550. doi: 10.3390/mi12050550 (PMC8151832; doi:10.3390/mi12050550)
Supplement: Supplementary file 1 [file micromachines-12-00550-s001.zip › micromachines-1176196-supplementary.pdf]

micromachines

Supplementary Materials

Evaluation of the Effects of Solvents Used in the Fabrication of Microfluidic Devices on Cell Cultures

Xiaopeng Wen [1], Seiichiro Takahashi [2], Kenji Hatakeyama [2] and Ken-ichiro Kamei [1,3,4,*]

[1] Institute for Integrated Cell-Material Sciences (WPI-iCeMS), Kyoto University, Yoshida-Ushinomiya-cho, Sakyo-ku, Kyoto 606-8501, Japan; bun.shouhou.4r@kyoto-u.ac.jp
[2] Incubation Center Organs On Chip Project, Ushio INC, 1-6-5 Marunouchi, Chiyoda-ku, Tokyo 100-8150, Japan; s.takahashi@ushio.co.jp (S.T.); k.hatakeyama@ushio.co.jp (K.H.)
[3] Wuya College of Innovation, Shenyang Pharmaceutical University, Liaoning 110016, China
[4] Department of Pharmaceutics, Shenyang Pharmaceutical University, Liaoning 110016, China
* Correspondence: kamei.kenichiro.7r@kyoto-u.ac.jp; Tel.: +81-75-753-9774

Citation: Wen, X.; Takahashi, S.; Hatakeyama, K.; Kamei, K-i. Evaluation of the effects of solvents used in the fabrication of microfluidic devices on cell cultures. *Micromachines* **2021**, *12*, 550. https://doi.org/10.3390/mi12050550

Academic Editor: Satoshi Fujita

Received: 24 March 2021
Accepted: 10 May 2021
Published: 12 May 2021

Publisher's Note: MDPI stays neutral with regard to jurisdictional claims in published maps and institutional affiliations.

Copyright: © 2021 by the authors. Licensee MDPI, Basel, Switzerland. This article is an open access article distributed under the terms and conditions of the Creative Commons Attribution (CC BY) license (http://creativecommons.org/licenses/by/4.0/).

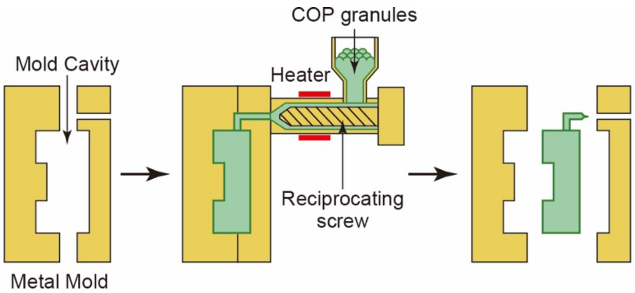

Figure S1. Metal molding process for fabricating the microfluidic structure of a COP-MPS.

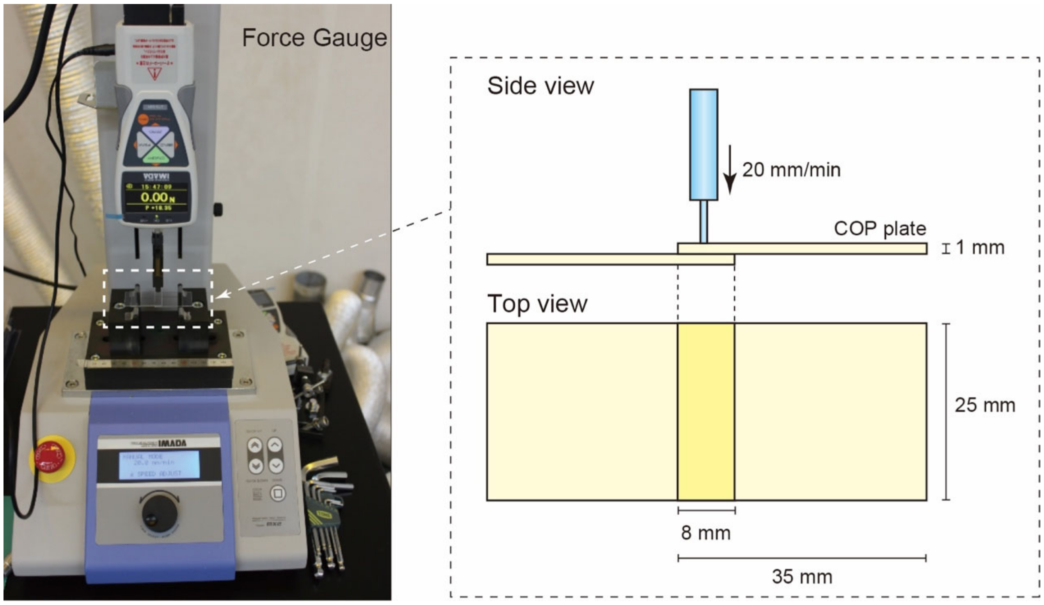

Figure S2. Experimental setup of the peeling test used to evaluate the bonding strength of the COP devices.

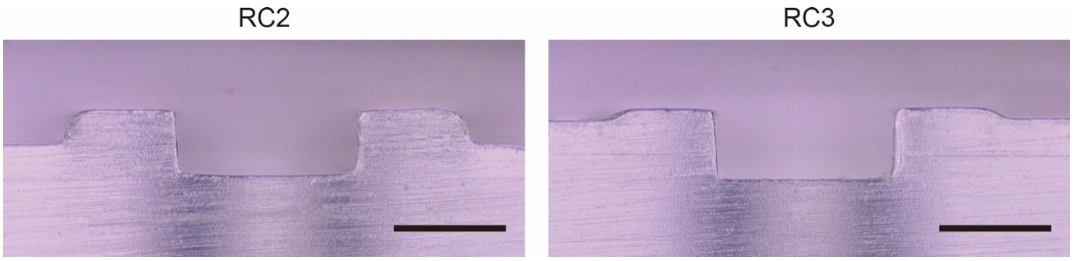

Figure S3. Cross-sections of the RC2 and RC3 COP microfluidic structures. The scale bar represents 500 μm.

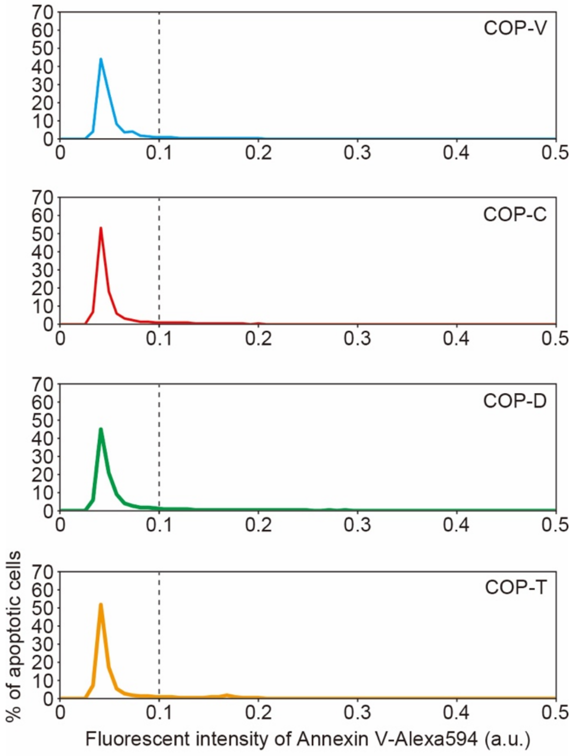

Figure S4. Histograms of the quantitative single-cell profiling of apoptotic cells stained with Annexin V labeled with Alexa 594 as the fluorescent dye. Stained cells showing greater than 0.1 of the fluorescence intensity of the Annexin V-Alexa 594 dye were defined as apoptosis “positive” cells.
